# Supplementary material for: The Protective Effects of Silkworm (Bombyx mori) Pupae Peptides on UV-Induced Skin Photoaging in Mice
Source: Foods. 2024 Jun 21;13(13):1971. doi: 10.3390/foods13131971 (PMC11241504; doi:10.3390/foods13131971)
Supplement: Supplementary file 1 [file foods-13-01971-s001.zip › foods-3009932-supplementary.pdf]

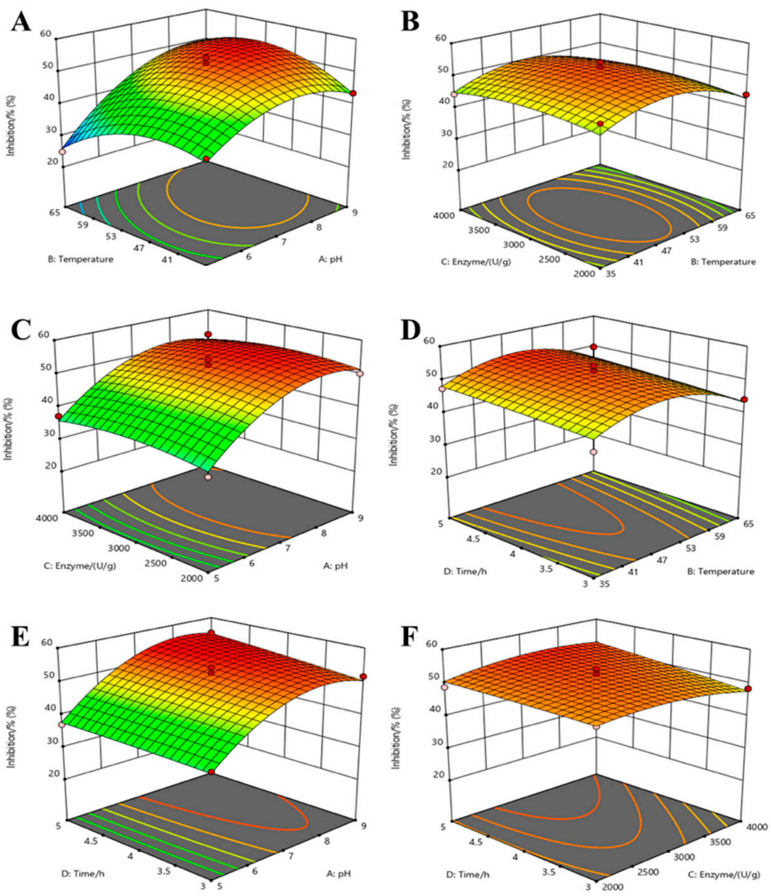

Figure S1. Response surface graph for Tyrosinase Inhibition Activity as a function of (a) temperature and pH; (b) temperature and enzyme dosage; (c) pH and enzyme dosage; (d) temperature and time; (e) pH and time; (f) enzyme dosage and time.

Table S1 Variance analysis results of the influence of enzymolysis parameters of the SPP.

**Source**	**Sum of Squares**	**df**	**Mean Square**	**F Value**	**p-value Prob>F**	
Model	1328.41	14	94.89	18.52	< 0.0001	significant
A	692.13	1	692.13	135.09	< 0.0001	
B	13.77	1	13.77	2.69	0.1233	
C	0.2885	1	0.2885	0.0563	0.8159	
D	19.87	1	19.87	3.88	0.0690	
AB	74.51	1	74.51	14.54	0.0019	
AC	0.5330	1	0.5330	0.1040	0.7518	
AD	3.85	1	3.85	0.7523	0.4004	
BC	0.0392	1	0.0392	0.0077	0.9315	
BD	0.1160	1	0.1160	0.0226	0.8826	
CD	5.21	1	5.21	1.02	0.3305	
A^2	328.73	1	328.73	64.16	<0.0001	
B^2	255.00	1	255.00	49.77	<0.0001	
C^2	24.95	1	24.95	4.87	0.0445	
D^2	0.2114	1	0.2114	0.0413	0.8420	
Residual	71.73	14	5.12			
Lack of Fit	65.07	10	6.51	3.91	0.1005	not significant
Pure Error	6.66	4	1.67			
Cor Total	1400.14	28				
